# 2-Amino-6-chloro-*N*-methyl­benzamide

**DOI:** 10.1107/S1600536813027827

**Published:** 2013-10-16

**Authors:** Yan-Hui Liang, Xiang-Dong Mei, Yao-Fa Li, Wen-Liang Pan, Jun Ning

**Affiliations:** a State Key Laboratory for Biology of Plant Diseases and Insect Pests, Institute of Plant Protection, Chinese Academy of Agricultural Sciences, Beijing 100193, People’s Republic of China; b Plant Protection Institute, Hebei Academy of Agricultural and Forestry Sciences, IPM Center of Hebei Province, Key Laboratory of Integrated Pest Management on Crops in Northern Region of North China, Ministry of Agriculture, Baoding 071000, People’s Republic of China

## Abstract

In the title compound, C_8_H_9_ClN_2_O, the dihedral angle between the benzene ring and the methyl­amide substituent is 68.39 (11)°. In the crystal, mol­ecules are linked by N—H⋯O hydrogen bonds, forming layers parallel to the *ab* plane.

## Related literature

For background information on substituted anthranilamides, see: Bharate *et al.* (2013 ▶); Gnamm *et al.* (2012 ▶); Lahm *et al.* (2005 ▶); Norman *et al.* (1996 ▶); Roe *et al.* (1999 ▶). For the synthesis, see: Witt & Bergman (2000 ▶); Coppola (1980 ▶).
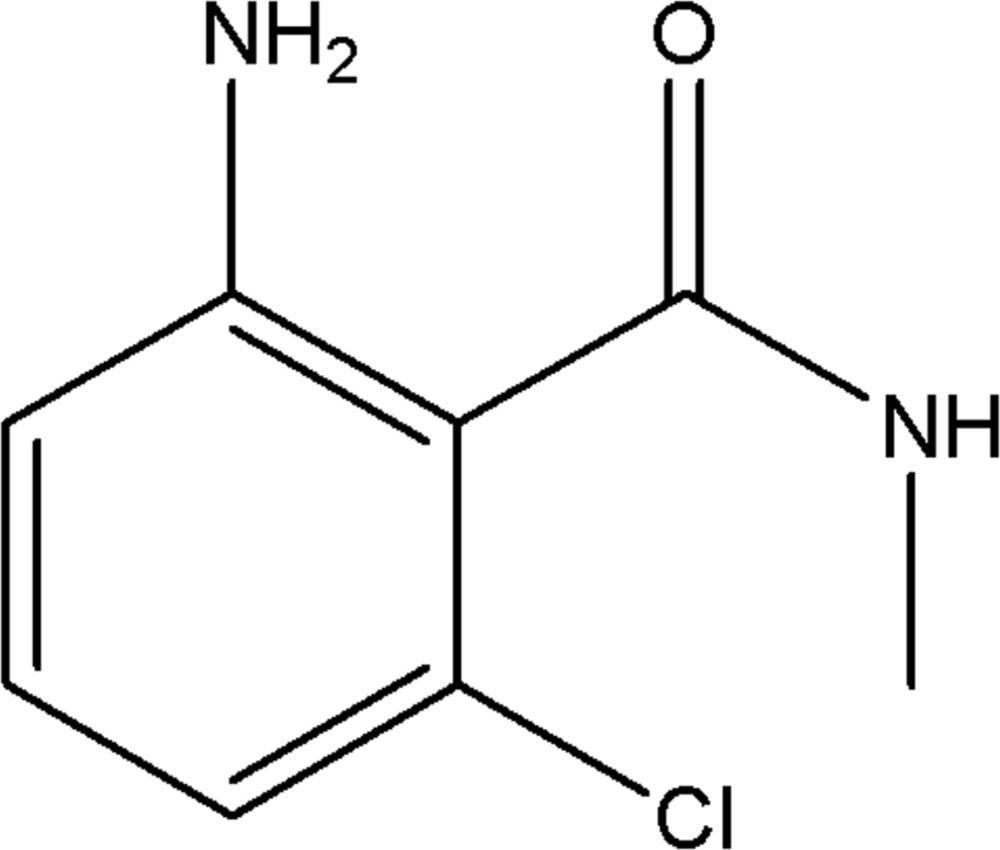


## Experimental

### 

#### Crystal data

C_8_H_9_ClN_2_O
*M* *_r_* = 184.62
Orthorhombic, 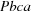

*a* = 9.2709 (19) Å
*b* = 11.812 (2) Å
*c* = 15.982 (3) Å
*V* = 1750.2 (6) Å^3^
*Z* = 8
Mo *K*α radiation
μ = 0.39 mm^−1^
*T* = 173 K
0.43 × 0.25 × 0.18 mm

#### Data collection

Rigaku MM007-HF CCD (Saturn 724+) diffractometer
Absorption correction: multi-scan (*CrystalClear*; Rigaku, 2007 ▶) *T*_min_ = 0.609, *T*_max_ = 1.000
3865 measured reflections
1528 independent reflections
1351 reflections with *I* > 2σ(*I*)
*R*_int_ = 0.042

#### Refinement

*R*[*F* ^2^ > 2σ(*F* ^2^)] = 0.065
*wR*(*F* ^2^) = 0.139
*S* = 1.17
1528 reflections
110 parameters
H-atom parameters constrained
Δρ_max_ = 0.26 e Å^−3^
Δρ_min_ = −0.26 e Å^−3^

### 

Data collection: *CrystalClear* (Rigaku, 2007 ▶); cell refinement: *CrystalClear*; data reduction: *CrystalClear*; program(s) used to solve structure: *SHELXS97* (Sheldrick, 2008 ▶); program(s) used to refine structure: *SHELXL97* (Sheldrick, 2008 ▶); molecular graphics: *Mercury* (Macrae *et al.*, 2006 ▶); software used to prepare material for publication: *SHELXL97*.

## Supplementary Material

Crystal structure: contains datablock(s) I, New_Global_Publ_Block. DOI: 10.1107/S1600536813027827/rz5085sup1.cif

Structure factors: contains datablock(s) I. DOI: 10.1107/S1600536813027827/rz5085Isup2.hkl

Supplementary material file. DOI: 10.1107/S1600536813027827/rz5085Isup3.cml

Additional supplementary materials:  crystallographic information; 3D view; checkCIF report

## Figures and Tables

**Table 1:** Hydrogen-bond geometry (Å, °)

*D*—H⋯*A*	*D*—H	H⋯*A*	*D*⋯*A*	*D*—H⋯*A*
N1—H1*B*⋯O1^i^	0.86	2.11	2.970 (3)	175
N2—H2⋯O1^ii^	0.86	2.04	2.895 (4)	172

Symmetry codes: (i) 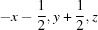; (ii) 
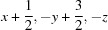
.

**Fig. 1.**
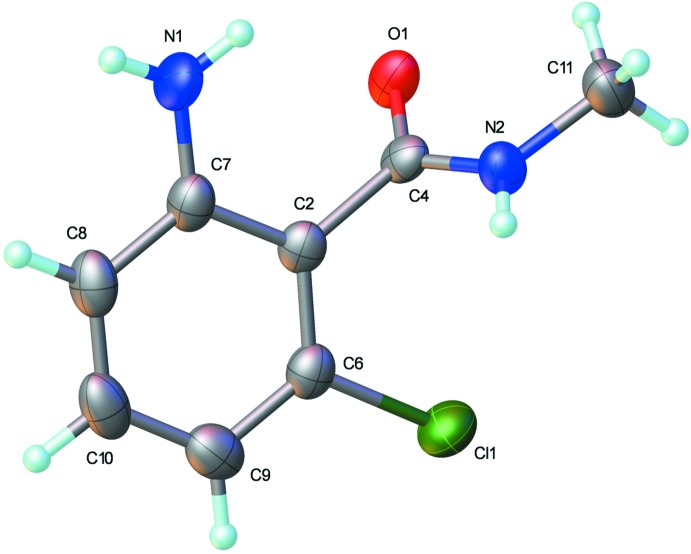
The molecular structure of the title compound showing 50% probability displacement ellipsoids.

## supplementary crystallographic information

### 1. Comment

Anthranilamide-based derivatives exhibit interesting biological activities such as antibacterial, antifungal, antiviral, antimalarial and insecticidal activities (Bharate *et al.*, 2013; Gnamm *et al.*, 2012; Lahm *et al.*, 2005; Norman *et al.*, 1996; Roe *et al.*, 1999). We report here the crystal structure of the title compound, 2-amino-6-chloro-*N*-methylbenzamide, an important organic intermediate in the synthesis of medicines, agricultural chemicals and animal drugs.

In the title compound (Fig. 1) the dihedral angle formed by the benzene ring and the methylamide substituent (r.m.s. deviation 0.0065 Å) is 68.39 (11)°. In the crystal structure, molecules are connected *via* N—H···O hydrogen bonds (Table 1) into layers running parallel to the *ab* plane.

### 2. Experimental

The title compound was prepared according to the literature method (Witt & Bergman, 2000) by stirring isatoic anhydride with aqueous methylamine. Isatoic anhydride was prepared by reaction of anthranilic acid with triphosgene in good yield (Coppola, 1980). The title compound (0.2 g) was dissolved in ethanol (50 ml) at room temperature. Colourless blocks were obtained through slow evaporation after two weeks.

### 3. Refinement

All H atoms were placed at calculated positions, with C—H = 0.93–0.98 Å, N—H = 0.86 Å, and refined as riding with *U*_iso_(H) = 1.2*U*_eq_(C, N) or 1.5*U*_eq_(C) for methyl H atoms.

### Crystal data

**Table:**

C_8_H_9_ClN_2_O	*F*(000) = 768
*M**_r_* = 184.62	*D*_x_ = 1.401 Mg m^−^^3^
Orthorhombic, *P**b**c**a*	Mo *K*α radiation, λ = 0.71073 Å
Hall symbol: -P 2ac 2ab	Cell parameters from 3698 reflections
*a* = 9.2709 (19) Å	θ = 1.3–27.5°
*b* = 11.812 (2) Å	µ = 0.39 mm^−^^1^
*c* = 15.982 (3) Å	*T* = 173 K
*V* = 1750.2 (6) Å^3^	Block, colourless
*Z* = 8	0.43 × 0.25 × 0.18 mm

### Data collection

**Table:**

Rigaku MM007-HF CCD (Saturn 724+) diffractometer	1528 independent reflections
Radiation source: fine-focus sealed tube	1351 reflections with *I* > 2σ(*I*)
Graphite monochromator	*R*_int_ = 0.042
Detector resolution: 28.5714 pixels mm^-1^	θ_max_ = 25.0°, θ_min_ = 2.6°
ω scans at fixed χ = 45°	*h* = −11→7
Absorption correction: multi-scan (*CrystalClear*; Rigaku, 2007)	*k* = −14→9
*T*_min_ = 0.609, *T*_max_ = 1.000	*l* = −10→19
3865 measured reflections	

### Refinement

**Table:**

Refinement on *F*^2^	Primary atom site location: structure-invariant direct methods
Least-squares matrix: full	Secondary atom site location: difference Fourier map
*R*[*F*^2^ > 2σ(*F*^2^)] = 0.065	Hydrogen site location: inferred from neighbouring sites
*wR*(*F*^2^) = 0.139	H-atom parameters constrained
*S* = 1.17	*w* = 1/[σ^2^(*F*_o_^2^) + (0.0417*P*)^2^ + 1.8852*P*] where *P* = (*F*_o_^2^ + 2*F*_c_^2^)/3
1528 reflections	(Δ/σ)_max_ < 0.001
110 parameters	Δρ_max_ = 0.26 e Å^−^^3^
0 restraints	Δρ_min_ = −0.26 e Å^−^^3^

### Special details

**Table:**

Geometry. All e.s.d.'s (except the e.s.d. in the dihedral angle between two l.s. planes) are estimated using the full covariance matrix. The cell e.s.d.'s are taken into account individually in the estimation of e.s.d.'s in distances, angles and torsion angles; correlations between e.s.d.'s in cell parameters are only used when they are defined by crystal symmetry. An approximate (isotropic) treatment of cell e.s.d.'s is used for estimating e.s.d.'s involving l.s. planes.
Refinement. Refinement of *F*^2^ against ALL reflections. The weighted *R*-factor *wR* and goodness of fit *S* are based on *F*^2^, conventional *R*-factors *R* are based on *F*, with *F* set to zero for negative *F*^2^. The threshold expression of *F*^2^ > σ(*F*^2^) is used only for calculating *R*-factors(gt) *etc*. and is not relevant to the choice of reflections for refinement. *R*-factors based on *F*^2^ are statistically about twice as large as those based on *F*, and *R*- factors based on ALL data will be even larger.

### Fractional atomic coordinates and isotropic or equivalent isotropic displacement parameters (Å^2^)

**Table:**

	*x*	*y*	*z*	*U*_iso_*/*U*_eq_	
Cl1	0.16414 (9)	0.67224 (7)	0.15558 (6)	0.0435 (3)	
O1	−0.2070 (2)	0.70451 (18)	0.04718 (16)	0.0403 (6)	
C2	−0.0247 (3)	0.8296 (2)	0.0969 (2)	0.0298 (8)	
N1	−0.1950 (3)	0.9652 (2)	0.0421 (2)	0.0435 (8)	
H1A	−0.2129	0.9255	−0.0017	0.052*	
H1B	−0.2227	1.0347	0.0402	0.052*	
C4	−0.0826 (3)	0.7415 (2)	0.0382 (2)	0.0302 (7)	
N2	0.0030 (3)	0.7101 (2)	−0.02413 (17)	0.0352 (7)	
H2	0.0870	0.7405	−0.0280	0.042*	
C6	0.0854 (3)	0.8068 (3)	0.1541 (2)	0.0332 (8)	
C7	−0.0872 (3)	0.9385 (3)	0.0972 (2)	0.0353 (8)	
C8	−0.0390 (4)	1.0177 (3)	0.1558 (2)	0.0398 (9)	
H8	−0.0809	1.0911	0.1568	0.048*	
C9	0.1338 (4)	0.8853 (3)	0.2115 (2)	0.0413 (9)	
H9	0.2095	0.8675	0.2493	0.050*	
C10	0.0685 (4)	0.9910 (3)	0.2123 (2)	0.0443 (9)	
H10	0.0982	1.0458	0.2522	0.053*	
C11	−0.0395 (4)	0.6261 (3)	−0.0866 (3)	0.0498 (10)	
H11C	−0.0103	0.6522	−0.1423	0.075*	
H11B	−0.1443	0.6160	−0.0851	0.075*	
H11A	0.0079	0.5538	−0.0742	0.075*	

### Atomic displacement parameters (Å^2^)

**Table:**

	*U*^11^	*U*^22^	*U*^33^	*U*^12^	*U*^13^	*U*^23^
Cl1	0.0438 (5)	0.0390 (5)	0.0476 (6)	0.0069 (4)	−0.0035 (4)	0.0071 (4)
O1	0.0297 (12)	0.0267 (12)	0.0646 (18)	−0.0043 (10)	0.0029 (11)	−0.0057 (12)
C2	0.0266 (15)	0.0251 (15)	0.038 (2)	−0.0052 (13)	0.0062 (14)	0.0020 (14)
N1	0.0440 (16)	0.0248 (14)	0.062 (2)	0.0047 (13)	−0.0059 (15)	−0.0056 (14)
C4	0.0281 (15)	0.0200 (14)	0.042 (2)	0.0012 (13)	−0.0025 (15)	0.0036 (14)
N2	0.0309 (13)	0.0341 (14)	0.0405 (17)	−0.0032 (12)	0.0040 (13)	−0.0069 (13)
C6	0.0306 (16)	0.0300 (17)	0.039 (2)	−0.0008 (14)	0.0073 (15)	0.0012 (15)
C7	0.0326 (16)	0.0270 (16)	0.046 (2)	−0.0029 (14)	0.0076 (16)	−0.0019 (15)
C8	0.048 (2)	0.0262 (17)	0.046 (2)	−0.0076 (16)	0.0130 (18)	−0.0030 (16)
C9	0.0365 (18)	0.051 (2)	0.036 (2)	−0.0082 (18)	0.0047 (16)	−0.0041 (18)
C10	0.0447 (19)	0.045 (2)	0.043 (2)	−0.0132 (18)	0.0148 (18)	−0.0141 (18)
C11	0.042 (2)	0.052 (2)	0.056 (3)	0.0000 (18)	−0.0020 (19)	−0.021 (2)

### Geometric parameters (Å, º)

**Table:**

Cl1—C6	1.749 (3)	C6—C9	1.379 (5)
O1—C4	1.242 (3)	C7—C8	1.397 (5)
C2—C6	1.395 (4)	C8—C10	1.381 (5)
C2—C7	1.411 (4)	C8—H8	0.9500
C2—C4	1.501 (4)	C9—C10	1.388 (5)
N1—C7	1.369 (4)	C9—H9	0.9500
N1—H1A	0.8601	C10—H10	0.9500
N1—H1B	0.8600	C11—H11C	0.9800
C4—N2	1.326 (4)	C11—H11B	0.9800
N2—C11	1.461 (4)	C11—H11A	0.9800
N2—H2	0.8600		
			
C6—C2—C7	118.4 (3)	C8—C7—C2	118.7 (3)
C6—C2—C4	122.5 (3)	C10—C8—C7	121.1 (3)
C7—C2—C4	119.1 (3)	C10—C8—H8	119.4
C7—N1—H1A	122.6	C7—C8—H8	119.4
C7—N1—H1B	117.4	C6—C9—C10	118.0 (3)
H1A—N1—H1B	115.8	C6—C9—H9	121.0
O1—C4—N2	123.0 (3)	C10—C9—H9	121.0
O1—C4—C2	120.2 (3)	C8—C10—C9	120.9 (3)
N2—C4—C2	116.7 (3)	C8—C10—H10	119.5
C4—N2—C11	122.8 (3)	C9—C10—H10	119.5
C4—N2—H2	118.6	N2—C11—H11C	109.5
C11—N2—H2	118.6	N2—C11—H11B	109.5
C9—C6—C2	122.9 (3)	H11C—C11—H11B	109.5
C9—C6—Cl1	117.7 (3)	N2—C11—H11A	109.5
C2—C6—Cl1	119.3 (2)	H11C—C11—H11A	109.5
N1—C7—C8	120.7 (3)	H11B—C11—H11A	109.5
N1—C7—C2	120.6 (3)		
			
C6—C2—C4—O1	111.3 (4)	C6—C2—C7—N1	179.7 (3)
C7—C2—C4—O1	−66.0 (4)	C4—C2—C7—N1	−2.8 (5)
C6—C2—C4—N2	−71.3 (4)	C6—C2—C7—C8	−1.6 (5)
C7—C2—C4—N2	111.3 (3)	C4—C2—C7—C8	175.9 (3)
O1—C4—N2—C11	−2.1 (5)	N1—C7—C8—C10	179.2 (3)
C2—C4—N2—C11	−179.4 (3)	C2—C7—C8—C10	0.5 (5)
C7—C2—C6—C9	1.1 (5)	C2—C6—C9—C10	0.5 (5)
C4—C2—C6—C9	−176.3 (3)	Cl1—C6—C9—C10	−178.0 (3)
C7—C2—C6—Cl1	179.6 (2)	C7—C8—C10—C9	1.2 (5)
C4—C2—C6—Cl1	2.2 (4)	C6—C9—C10—C8	−1.7 (5)

### Hydrogen-bond geometry (Å, º)

**Table:**

*D*—H···*A*	*D*—H	H···*A*	*D*···*A*	*D*—H···*A*
N1—H1*B*···O1^i^	0.86	2.11	2.970 (3)	175
N2—H2···O1^ii^	0.86	2.04	2.895 (4)	172

Symmetry codes: (i) −*x*−1/2, *y*+1/2, *z*; (ii) *x*+1/2, −*y*+3/2, −*z*.
